# Social organization and physical environment shape the microbiome of harvester ants

**DOI:** 10.1186/s42523-025-00390-3

**Published:** 2025-03-19

**Authors:** Denisse Alejandra Gamboa, Peter J. Flynn, Eva Sofia Horna-Lowell, Noa Pinter-Wollman

**Affiliations:** 1 Department of Ecology and Evolutionary Biology, University of California Los Angeles, Los Angeles, CA 90095 USA; 2 Department of Organismic and Evolutionary Biology, Harvard University, Cambridge, 02138 USA; 3 San Diego Natural History Museum, Balboa Park, San Diego, 92101 CA USA

**Keywords:** Microbiome, Social organization, Physical environment, Harvester ant, Veromessor Andrei

## Abstract

All animals harbor microbiomes, which are obtained from the surrounding environment and are impacted by host behavior and life stage. To determine how two non-mutually exclusive drivers - physical environment and social organization - affect an organism’s microbiome, we examined the bacterial communities within and around nests of harvester ants (*Veromessor andrei*). We collected soil and nest content samples from five different ant nests. We used 16S rRNA gene sequencing and calculated alpha and beta diversity to compare bacterial diversity and community composition across samples. To test the hypotheses that physical environment and/or social organization impact ant colonies’ community of microbes we compared our samples across (i) sample types (ants, brood, seeds and reproductives (winged alates), and soil), (ii) soil inside and outside the nest, and (iii) soil from different chamber types. Interestingly, we found that both the environment and social organization impact the bacterial communities of the microbiome of *V. andrei* colonies. Soil from the five nests differed from one another in a way that mapped onto their geographical distance. Furthermore, soil from inside the nests resembled the surrounding soil, supporting the physical environment hypothesis. However, the bacterial communities associated with the contents within the nest chambers, i.e., ants, brood, seeds, and reproductives, differed from one another and from the surrounding soil, supporting the social organization hypotheses. This study highlights the importance of considering environmental and social factors in understanding microbiome dynamics.

**Supplementary Information:**

The online version contains supplementary material available at 10.1186/s42523-025-00390-3.

## Background

Animals are associated with and are colonized by communities of microorganisms that are shaped by biotic and abiotic conditions, known collectively as the microbiome. In addition to vertical and horizontal acquisition, animals obtain microbes from the environment, and their behavior and life stage have a large impact on their microbiome. Throughout their lives, animals are colonized by microbes from their surroundings [1–3]. Because microbes are acquired in various ways, composition of microbial communities depends on how they are obtained or transmitted. Studies in humans and non-human primates suggest that the surrounding environment including habitat, diet, or social group can significantly influence microbiome composition [4–6]. The composition of the gut microbiome varies across captive, urban, and rural environments in many organisms such as Ring-tailed lemurs [7], Tasmanian devils [8], deer mice [9], water dragons [10], coyotes [11], beetles [12], carpenter bees [13], and honey bees [14]. In addition to the environment, host behaviors significantly impact host-microbiome dynamics. For example, communal nesting in four-toed salamanders (*Hemidactylium scutatum*) increases the transmission of beneficial, antifungal bacteria, enhancing hatchling survival compared to solitary nests [15, 16]. Furthermore, coprophagy, a behavior involving the consumption of feces, regulates gut microbiota in vertebrates and invertebrates [17, 18]. Thus, both the physical environment and the behavior of an individual impact its microbiome.

Host-associated microbiome studies often consider microbial communities inside or on the surface of the organism, however, many animals occupy stable burrows or construct nests [19]. Because of the large amount of time that animals spend in their nests and burrows [20, 21], the microbial communities of these built structure comprise much of the microbial communities that animals are exposed to through the physical environment. Indeed, the type of building materials used in nest construction can affect the health of the animals that build them, if materials such as resin and leaves with anti-bacterial properties are incorporated into the nest [22]. Despite this potentially large impact of nest microbial communities on its inhabitants, little is known about the relationship between the microbiome inside a nest and the microbiome of its inhabitants. Environmentally acquired microbes tend to be ephemeral and not host-specific due to the functional redundancy of bacterial species and the changing environmental conditions that both hosts and their microorganisms are exposed to (e.g., temperature, humidity, nutrients) [23]. Indeed, the microbiome of animals is often determined by the environment in which they live. For example, when the cuticular microbiomes of two arboreal ant species were compared, the physical location of their nest was a better predictor of their microbiome composition than the species of ant [24]. Similarly, when comparing the gut microbiome of weaver ants (*Oecophylla smaragdina*) from forest and urban environments, forest colonies had an increased abundance of Acetobacteraceae compared to urban colonies [25]. Therefore, the relative impact of the environment on the microbiome of an animal is important to consider, especially for animals whose environment is an integral component of their lives, such as soil-nesting ants. Microbial diversity and biomass in soils has been linked to a wide range of soil properties including factors that change with soil depth [26] such as soil pH, soil organic carbon, and oxygen [27, 28]. Therefore, we expect that if the microbiome of animals that live in soil is impacted by the environment, such subterranean animals will have microbiomes that mirror the soil’s, including decreased microbial diversity with depth.

The behavior of an animal and the social organization of a population can impact the microbiome of an animal and the microbiome can provide information specific to the host. For example, in spotted hyenas, microbiome varies with sex and age-class and it is specific to individuals [29]. Further, the gut microbiome composition of individual chimpanzees from the same communities are similar due to their shared diets but long-term immigrants into the population show distinct gut microbiome composition, suggesting that immigrant individuals retain characteristics of their original community’s gut microbes, despite moving to a new environment [30]. In social insect colonies individuals perform different behavioral tasks, which influence and structure the microbiome composition of individuals within colonies. For example, honeybee workers that perform different behavioral tasks, such as foraging, or nursing, show differences in gut bacterial community composition [31] and the gut microbiome can influence the onset of certain tasks, such as foraging [32]. Additionally, there are differences in the gut microbiome composition of reproductives and non-reproductive workers in termites [33, 34], honey bees [35, 36], and ants [37, 38]. Thus, the behavioral role of individuals can have a strong impact on their microbiome.

**Fig. 1:**
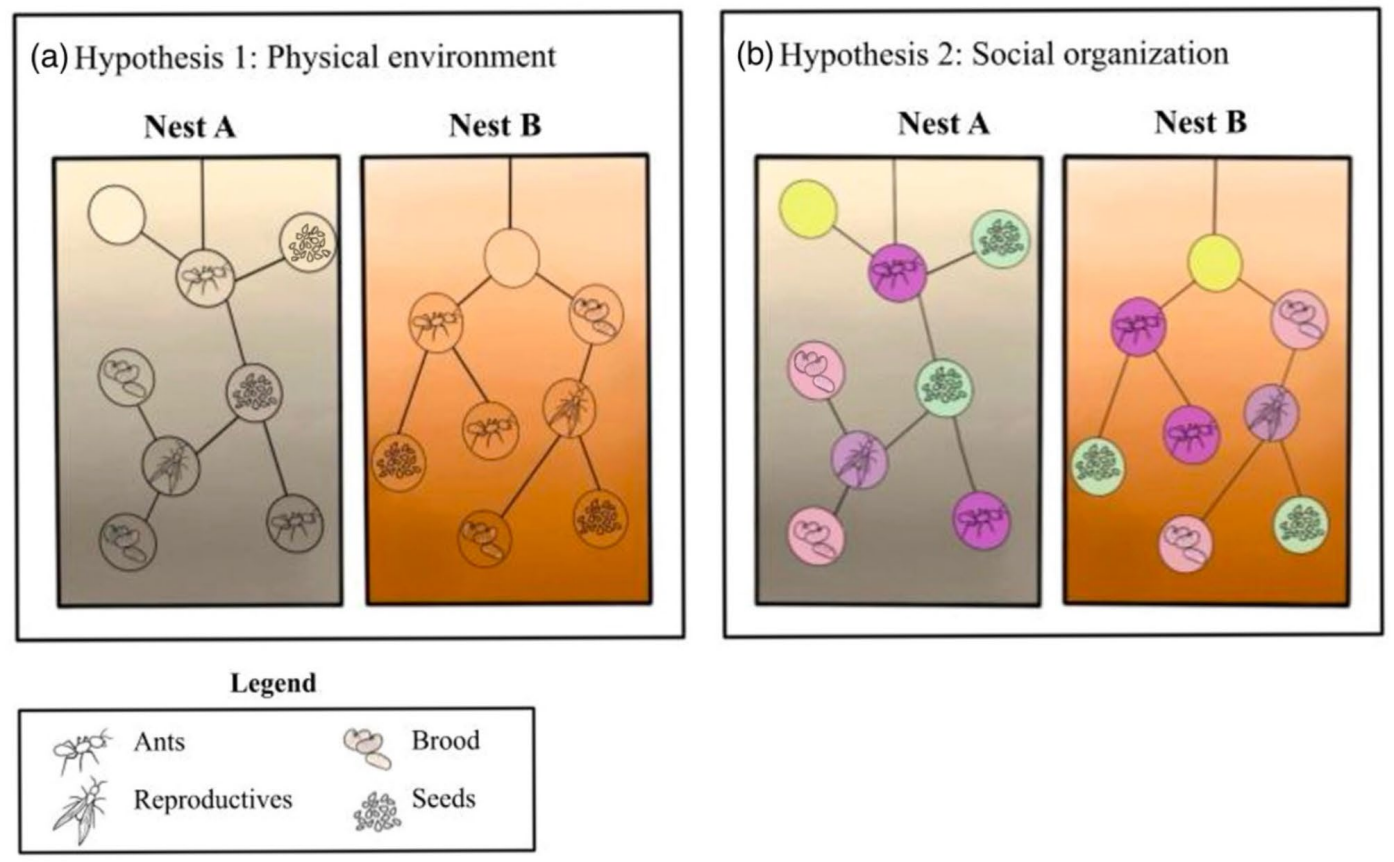
Visualization of hypotheses. (**a**) The physical environment determines microbial community structure. We expect the contents within the nest chambers (ants, reproductives, brood, and seeds) to have similar microbiome composition to the surrounding soil and that nests will differ from one another in their microbiome - based on differences in the surrounding soil. (**b**) Social organization determines microbial composition. We expect the microbiome composition of contents within the nests chambers to differ across chambers regardless of the microbial composition of the surrounding soil. The colors of the circles represent different microbiome compositions of the nest chambers. The background colors represent the microbiome composition of the surrounding soil

Ants are highly social animals that shape the environment in which they live - their nest. Ants create nests, for example, by connecting leaves with silk [39], digging through wood [40], excavating soil [39] which alters soil distribution [41], among other means. The nest microbiome interacts with the ant microbiome. For example, the cuticular microbiome of two arboreal Amazon ant species overlaps with the bacterial microbes found within their nests [24]. Most studies of ant microbiomes have focused on the gut microbiome, showing that ant species differ in the densities of bacterial communities in the gut according to diet type [42] and that ants can benefit from their microbiome via nutrient acquisition and defense against pathogens [42, 43]. However, to our knowledge, the role of the nest in shaping the microbiome of ant colonies has only been explored in arboreal ants that occupy exiting tree cavities [44] and not in subterranean ants that construct and shape their own nest. In lab experiments, each nest region was found to have a different chemical signature that reflects the individuals that occupy that area [45]. Thus, it is likely that a similar relationship between the structure of subterranean nests and the materials inside each chamber shape the microbiome of a subterranean ant colony and its nest, as seen in arboreal ants [44]. Across ant species, behavioral tasks occur at specific locations within a nest, such that when not foraging, foragers are found near the entrance of the nest and brood nurses are found in the center, where the brood is located [45–48]. This spatial division of labor can structure the microbial composition of individuals within colonies of ants. The relationship between social organization and spatial position suggests that the physical environment and social organization combine to influence the microbiome of ant colonies. For example, nest chambers of ants might differ in their microbiome composition based on the behavioral tasks performed in them. Such potential differences in chamber microbiome can be driven by the chambers’ content (e.g., the seeds or the brood) or by the ants that tend to the chamber material (Fig. 1). Furthermore, the microbiome found inside ant nests might differ from the surrounding soil, just as plant composition on nest mounds of ants differs from the surrounding environment [49].

Colonies of the harvester ants, *Veromessor andrei*, live in grasslands, where they turn and aerate the soil, redistributing nutrients and potentially creating favorable conditions for microorganisms within and around their subterranean nests [49–52]. Colonies of *V. andrei* are large, reaching sizes of tens of thousands of workers [53], and one queen, i.e., the colonies are monogynous [53, 54]. They nest in grasslands habitats, where their primary diet is seeds, which workers gather by following long (up to tens of meters) foraging trails [54, 55]. *Veromessor andrei* nests provide an excellent opportunity to examine the effects of the physical environment and social organization on the microbiome of the colony because of the strong effects that nest structure has on colony behavior. Nests of *V. andrei* are comprised of chambers connected with tunnels and the connectivity among nest chambers, and especially that of the entrance chamber, affects the speed of foraging recruitment [56]. The impact of nest structure on foraging behavior happens most likely through the impact of the nest structure on interactions among ants within the nest [57], which regulate foraging activity in other harvester ants [58–60]. Nest structure can potentially further segregate behavioral tasks such as brood care and food storage. However, it is not known how nest structure and social organization combine to impact the microbiome of harvester ant colonies.

To determine the roles of the physical environment and social organization in structuring an organism’s microbiome we examined the bacterial communities of the microbiome within and around nests of *V. andrei*. Specifically, we test two non-mutually exclusive hypotheses: (1) the physical environment determines microbial community structure; and (2) social organization determines microbial composition (Fig. 1). We predict that if the physical environment influences microbial communities (1) the bacterial communities associated with the content of the nest (like ants, seeds, brood, etc.) will be similar to the bacterial communities in the surrounding soil; (2) the bacterial communities in the soil inside nest chambers will not differ from those in the surrounding soil; (3) bacterial diversity in nest chambers will decrease with nest depth, similarly to the relationship between depth and microbiome in soils [26]; and (4) nests in different locations will have different bacterial diversity because soils change their microbial composition and diversity spatially [27, 28] (Fig. 1a). We predict that if social organization influences microbial communities of ant nests (1) nest content (like ants, seeds, brood etc.) will differ in their bacterial composition according to their biological classification and will be different from the bacterial composition of the surrounding soil; (2) bacterial diversity of chamber soil will differ across chambers according to the content found in them, regardless of the bacterial communities in the surrounding soil; (3) bacterial diversity of chamber soil will differ from the bacterial diversity of the surrounding soil; and (4) bacterial composition of soil inside nest chambers will be conserved by the content of the chamber in a way that is consistent across different nests (Fig. 1b).

## Methods

### Study site and sample collection

**Fig. 2:**
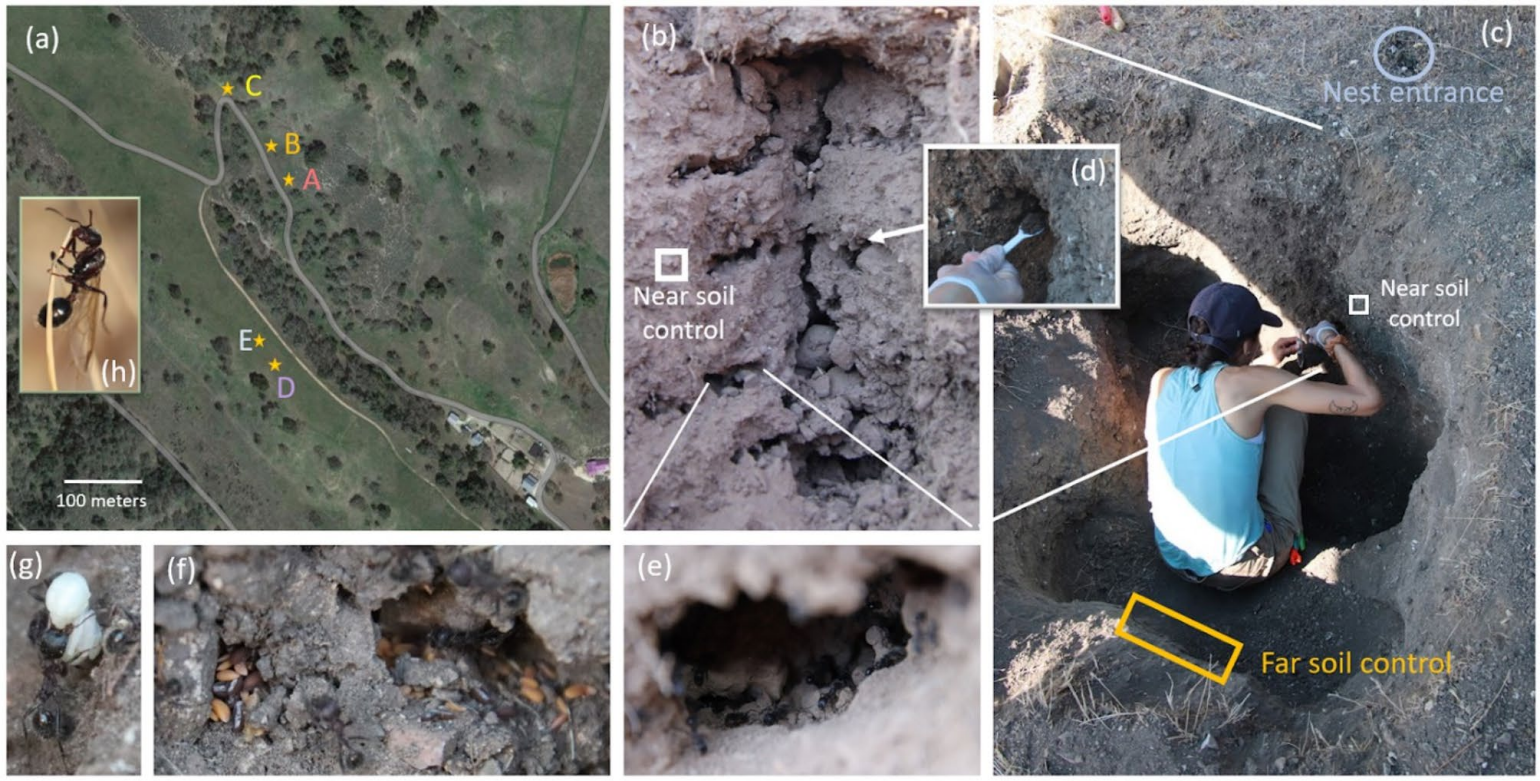
Sample collection. (**a**) Map of the study site with the locations of the five nests that we excavated indicated with orange stars and the letter ID of each colony, which is colored according to their representation in Fig. 6. (**b**) the excavated nest of colony D; the white box indicates the approximate location where we sampled ‘near soil control’ for the chamber immediately to the right of the white box. (**c**) EHL crouching in the trench we dug to reach the nest chambers from the side, sampling soil from one of the chambers. A white box indicates the general area from which a ‘near soil control’ sample would be taken and the yellow rectangle shows the approximate location where ‘far control soil’ samples were collected. The entrance of the nest is indicated at the top right in a gray circle. (**d**) sampling soil from inside a chamber with a plastic spoon. We collected each chamber’s content, including (**e**) ants, (**f**) seeds, (**g**) brood, and (**h**) reproductives. All photo credits: Noa Pinter-Wollman

To examine the microbiome of ant nests’ soil and of the content of the nests, we collected samples from five colonies of *V. andrei* in May 24–29, 2021 from a serpentine grassland at the Sedgwick Natural Reserve in southern California, US. Sedgwick Reserve is home to a thriving *V. andrei* population (> 100 colonies) and we selected five colonies that could be easily accessed, were far from other ant nests, to reduce any negative impact of excavation on other colonies, and whose nests were off the road - so that nest excavation would not disrupt access (Fig. 2a). To access the nest content, we first dug a trench approximately 1–1.5 m away from the nest entrance, using a tractor fitted with a post hole digger, pickaxes, and shovels. Once the trench was established, we began digging towards the nest until we reached a chamber from its side and sampled its content, as detailed below. Once we completed sampling a chamber, we continued excavating in the direction of the tunnels leading out of the chamber, until we found another chamber and sampled it too. We proceeded to excavate and sample from all nest chambers until we could not find any more new chambers that were not sampled (Fig. 2b). When we reached a chamber, we collected with gloves or soft tweezers (sterilized with ethanol) samples of its content, which included ants, brood, seeds, and reproductives (defined as male and unmated female winged alates and not the founding queen) (Fig. 2e-h). Most chambers included ants, but not all chambers included brood, seeds, and reproductives. We placed each type of sample in a separate, labeled, 15 ml tube. After sampling the chamber’s content, we used a small disposable plastic spoon to collect soil from inside the excavated chamber (Fig. 2d), referred to later as ‘nest soil’. Nest chamber soil samples were classified into ‘chamber types’ according to the chamber content (e.g., if they had ants inside, they were considered ‘ant’ chamber type). If a chamber had more than one type of content (e.g., both brood and reproductives were found in the same chamber) the chamber was assigned a type based on all the material in it (e.g., brood + reproductives). We then (using a new disposable plastic spoon) sampled soil from a location outside the nest, within approximately 5 cm of the excavated chamber, and at the same depth as the chamber, which we referred to as ‘near soil control’ (Fig. 2b, c). We recorded the depth of the chamber from the ground surface using a measuring tape. Once all chambers were excavated, we obtained the ‘control far soil’ samples by collecting soil from the side of the trench that was opposite the nest (Fig. 2c). We used a measuring tape to sample soil from depths that matched those of the chambers we excavated. Thus, each chamber had several associated samples - three soil samples (from inside the chamber, near the chamber, and far (~ 1-2m) from the chamber - at the same depth) and samples of the content of the chamber (ranging from 1–3 additional samples - depending on the content we found). Each day we excavated one nest, with the first four nests (A, B, C, and D) excavated on consecutive days (May 24–27, 2021) and the fifth one (E) collected after a one-day break (on May 29th, 2021). Around noon and at the end of each day, around 5pm, we placed the samples we collected in a freezer at the field station. Nests differed in depth and number of chambers. All samples were transferred in a cooler to the UCLA campus (approximately a 2-hour drive from the field site), where they were stored in a -20 freezer until processing.

### Sample processing

After collection, we stored and kept the samples at -20 °C until extractions. To prepare the soil samples for extractions we weighed, using a microscale, up to 250 mg of soil and placed the soil into sterile 1.5 ml tubes. To prepare the non-soil samples (ants, seeds, brood etc.) for extractions we first washed ant workers and alates three times: first in 70% ethanol, then in 5% bleach, and then with sterile deionized water. We placed each washed sample in a sterile 1.5 ml tube and used a sterile pestle (provided in the kit by Qiagen), that specifically fits in 1.5 ml tubes, to crush the ants. Seed and brood samples were crushed but not washed. Brood samples were not washed because the bleach and alcohol used in the wash protocol would have destroyed the samples. Seeds were not washed because we were interested in quantifying the microbial communities on their exterior. We added 800 µl of Solution C1 from the Qiagen DNEasy PowerSoil Pro kit, containing SDS for cell lysis to all samples (including soil) following manufacturer’s instructions. To facilitate cell lysis, we vortexed and left the samples overnight in an incubator at 56 °C. Microbial DNA was extracted using the same Qiagen DNEasy PowerSoil Pro kit following manufacturer’s instructions. After DNA extraction, 285 samples were sent for sequencing at the UCLA Microbiome Center for 16 S rRNA gene amplification and library sequencing. Amplicon sequencing of the bacterial community was performed using the V4 region of the 16 S rRNA gene using the primers 515 F (59-GTGCCAGCMGCCGCGGTAA-39) and 806R (59-GGACTACHVGGGTWTCTAAT-39) following the Earth Microbiome Project (EMP) protocol [61, 62].

We collected and sequenced 285 samples (see data in the Github repository: https://github.com/DAlejandraG/nest-microbes). The 16 S rRNA gene amplicon sequencing raw reads are available from NCBI via BioProject record PRJNA1147938. The raw dataset contained a total of 4,884,506 reads. We rarefied the dataset at a sampling depth of 3,618 and retained 955,152 features (25.73%) after refraction with a total of 264 samples (92.65%) after 22 samples were removed. We then removed 21 sample types that were obtained for only some nests, or did not have a large enough sample size to include in the analysis (e.g., entrance soil and soil from the mound). Lasty, we removed four samples due to errors in labeling during sample collection or during sample extraction. Code for this data cleaning is available on Github: https://github.com/DAlejandraG/nest-microbes.

### Quantifying bacterial community diversity

To determine the bacterial community composition of each sample type (soil, seeds, brood, adult ants), bioinformatics were conducted using QIIME2 version 2024.2.4 [63]. Initial raw sequence data underwent demultiplexing and quality filtering with the q2-demux plugin, followed by denoising using DADA2 [64] through the q2-dada2 plugin. Amplicon Sequence Variants (ASVs) were aligned using MAFFT [65] via q2-alignment, and a phylogeny was constructed with FastTree2 [66] through q2-phylogeny. ASVs were assigned taxonomy using the q2-feature-classifier [67] classify-sklearn naive Bayes taxonomy classifier, referencing the Silva 13_8 99% OTU database [68]. ASVs are used as a proxy for bacterial species and are similar to OTUs (operational taxonomic units) but at a finer-scale resolution (100% similarity). Once the quality filtering steps were completed, we estimated refraction, alpha and beta diversity measures using q2 diversity based on ASVs. We created a summary feature table (see Github: https://github.com/DAlejandraG/nest-microbes) with information on how many sequences are associated with each sample. To create relative abundance plots at the phylum, order, family, and genus taxonomic levels, and assess species composition, we exported the feature table and used the ‘*phyloseq*’ package in R [69]. To further examine the abundance and phylogenetic relationship among the most abundant ASVs in the ant and soil samples, we pruned the phylogeny using the drop.tip function from the ape R package [70], retaining only the ASVs detected in each sample type. The ComplexHeatmap package in R [71] was used to generate heatmaps of the top 20 most abundant ASVs for ant and nest soil samples. Additionally, Similarity Percentage Analysis (SIMPER) was conducted to identify the specific contributions of ASVs to the composition of bacterial communities across ant and soil samples. The analysis was performed in R using the Vegan package [72] with 999 permutations.

To determine how the nest-associated bacterial communities are influenced by the physical and social environment, we examined diversity within (alpha diversity) and among (beta diversity) samples. The input for all diversity measures was ASVs. Alpha diversity indices provide information regarding the number of microbial taxa in a single sample. The alpha diversity indices we used include:

1. Shannon’s index - describes how evenly species are distributed, independent of species richness [73, 74]. A high Shannon index indicates more species diversity whereas a value of zero indicates that fewer species are present in the sample.
2. Faith’s phylogenetic diversity - a weighted measure of richness that describes the amount of the phylogenetic tree that is covered by the communities, i.e. more evolutionary branches would result in greater diversity [75].
3. Pielou’s evenness - provides information about the relative abundance of species in a sample, i.e., if some species are dominating others or if all species have similar abundances [76].
4. Observed amplicon sequence variants (Observed ASVs) - the number of observed unique sequences that are present in the sample [64].

Beta diversity provides information about the differences in bacterial community composition among multiple samples, classifying samples into groups according to similarities in their bacterial composition based on sequence abundances or the presence or absence of sequences [77]. Here we used the Bray-Curtis dissimilarity index as a beta diversity measure of compositional dissimilarity among bacterial communities [78]. We measured beta diversity differences between samples using a permutational multivariate analysis of variance (PERMANOVA) on Bray-Curtis dissimilarity matrices. Principal Coordinate Analysis (PCoA) ordination was calculated based on these matrices using the Adonis [79] and Vegan package [72], with 999 permutations for the PERMANOVA. The resulting PCoA plots were visualized using ggplot2 [80]. We performed the PERMANOVA on Bray-Curtis distances calculated from the rarefied dataset to test for dissimilarities in bacterial community composition among samples based on the ASVs. This analysis tested for differences in beta diversity among all sample types, all soil types, and nest soil samples.

### Statistical analysis

All analysis was conducted in R version 4.3.2 [81] and all of the best-fitting models met the required statistical assumptions – examined using the check_model() function in the ‘performance’ package [82].

*All sample types*: To determine if alpha diversity differed across all sample types, we ran four linear models (LM) - one for each of the four alpha diversity measures (Shannon, Faith’s phylogenetic diversity, Pielou’s evenness, and ASV richness) as the response variable. The explanatory variable was the type of sample (ants, seeds, reproductive, brood, or soil). We used the lm() function in R [81] for these models. For post-hoc comparisons of bacterial diversity among sample types, we used a post hoc Tukey test by applying the Tukey HSD() function in R [81]. We further examined PCoA plots and used a PERMANOVA to examine beta diversity across sample types.

*All soil samples*: To determine if alpha diversity changed with soil depth and differed across locations, we ran linear models (LM) implemented as detailed above. In each model one of the four alpha diversity measures (Shannon index, Faith’s phylogenetic diversity, Pielou’s evenness, and ASV richness) was the response variable. The explanatory variables included: depth, nest ID, and soil type (chamber soil, control near, and control far). For post hoc tests we used the package ‘emmeans’ [83]. We used a model selection approach to determine which interaction terms to include in our final statistical model. We ran each model with either no interactions among soil type, depth, and nest ID; with the three-way interaction among the three variables; and three additional models with just one interaction each between a different pair of variables each time, totaling five statistical models per alpha diversity measure. We then compared the models using AIC [82] and selected the best fit model, i.e., the one with the lowest AIC score. The best fit models for all diversity measures included no interaction terms among the explanatory variables. For specific comparisons of bacterial diversity among soil types, we used a post hoc Tukey test [83]. We further examined PCoA plots and used a PERMANOVA to examine the beta diversity among soil samples and the five different nests. For specific comparisons of bacterial diversity among soil types, we used pairwise PERMANOVA tests by applying the pairwise.adonis( ) function in the package ‘pairwiseAdonis’ [79]. To assess differences in the amount of dispersion, we conducted a permutational analysis of multivariate dispersions (PERMDISP) by applying the betadisperser( ) and permutest( ) functions in the package ‘Vegan [72].

*Nest soil samples*: To determine if the bacterial composition in the soil inside nest chambers differed based on chamber type (i.e., the content found in the chamber: ants, seeds, reproductives, and brood), we ran four linear models (LM) and post hoc tests as detailed above. In each model, the response variable was one of the four diversity indices (Shannon index, Faith’s phylogenetic diversity, Pielou’s evenness, and ASV richness) and the explanatory variables included: nest ID, sample depth, and chamber type (based on the content listed above). We used the same model selection approach detailed above [82] and if the best fit model included an interaction term, but the collinearity was very high (VIF > 10), we removed the interaction term. Due to high collinearity among terms in the models, we ended up keeping only models with no interaction terms. We further examined PCoA plots and used a PERMANOVA to examine beta diversity across chamber types.

Data and code are available in the Github repository: https://github.com/DAlejandraG/nest-microbes.

## Results

### All sample types

**Fig. 3:**
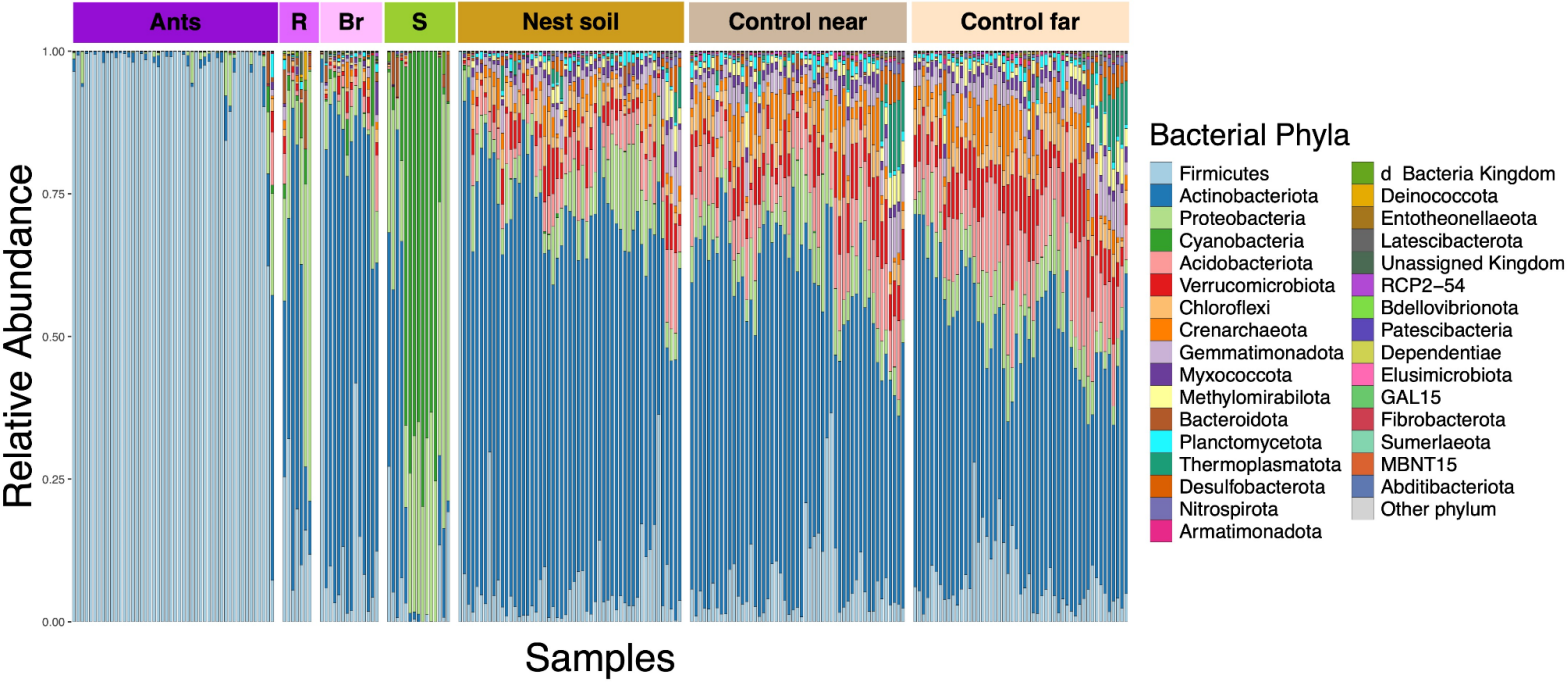
Relative abundance of bacterial phyla ordered by sample type: ants, reproductives (R), brood (Br), seeds (S), nest soil, control near soil, control far soil. Each vertical bar is an individual sample with color indicating the bacterial phyla according to ASV. The sampling depth was 3618 reads. For abundance plots by class, order, family, and genus see Figs. S1, S2, S3, and S4 respectively

**Fig. 4:**
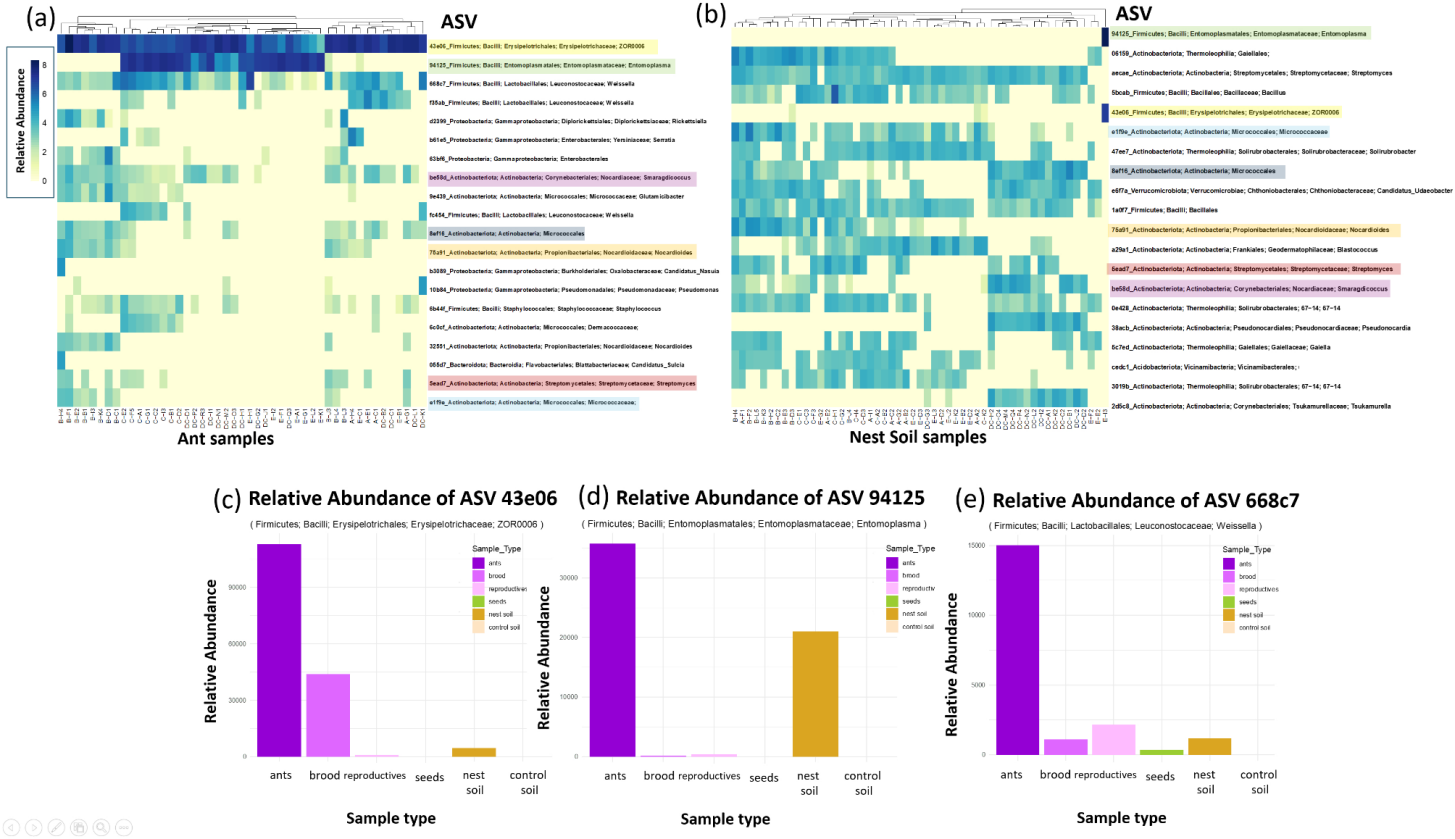
Analysis of ASVs in soil and ants. (**a**) Heatmap of the abundance of the top 20 ASVs for all ant samples and (**b**) top 20 ASVs for all nest soil samples. Names of ASVs which are the same between ant and nest soil samples are highlighted in the same color in (**a**) and (**b**). Color in the heatmaps indicates relative abundance – see color bar to the left. Heatmaps are arranged by overall abundance of ASVs – with the most abundant ASV at the top row of each heatmap and the lowest abundance ASV at the bottom row. (**c**) Relative abundance of the most abundant ASV (43e06) across all sample types. (**d**) Relative abundance of second most abundant ASV (94215) across all sample types. (**e**) Relative abundance of the third most abundant ASV (668c7) across all sample types

**Fig. 5:**
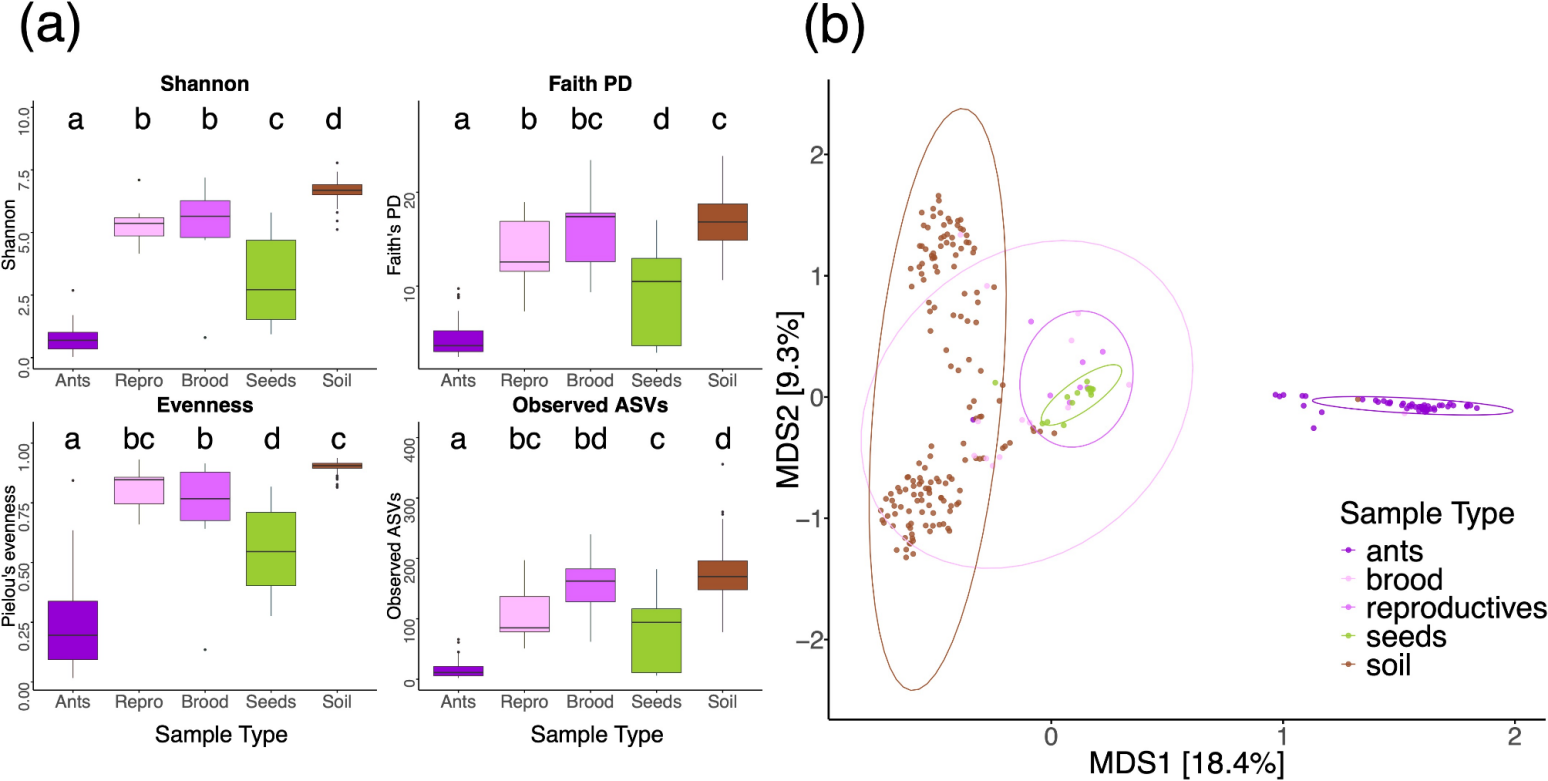
Alpha and beta diversity measures by sample type: ants, reproductives (repro), brood, seeds, and soil. (**a**) Box plots of microbiota alpha diversity measures (Shannon, Faith’s phylogenetic distance (PD), Pielou’s evenness and Observed ASVs) by sample types. Here, and in all following figures, boxes indicate interquartile ranges, lines inside the boxes denote medians, whiskers extend to 1.5 times the interquartile range, and dots are outliers. Boxes that do not share letters are statistically different according to a post hoc Tukey test (p-value < 0.05). (**b**) Beta diversity Principle coordinate analysis (PCoA) from Bray-Curtis dissimilarity matrix by sample types. Each point represents one sample and is color coded by sample type. The closeness of points indicates high community similarity

The alpha, and beta diversity of all samples differed significantly by sample type, supporting the social organization hypothesis. Ants, reproductives, brood, seeds, and soil had different ASV compositions, regardless of which taxonomic level we examined (Figs. 3, S1, S2, S3 and S4). The top three ASVs varied in relative abundance across sample type (Fig. 4). Furthermore, ants, reproductives, brood, seeds, and soil significantly differed in alpha diversity, calculated based on ASVs, regardless of which diversity measure we examined (Table 1; Fig. 5). A post hoc Tukey test showed that ant samples had the lowest, and soil samples had the highest, alpha diversity compared to all other sample types, across all measures of alpha diversity. Brood and reproductives did not differ significantly in their alpha diversity across all diversity measures. Finally, seeds and reproductives showed significant differences in the Faith’s Phylogenetic distance measure (Fig. 5a).

The principal coordinate analysis (PCoA) demonstrated that the samples’ ASVs clustered by ‘sample type’ (Fig. 5b). ‘Sample type’ explained a significant amount of variation in the dataset, explaining approximately 22.42% of the total variation (PERMANOVA: F_DF = 4_ = 16.91, R^2^ = 0.22, p-value = 0.001). Pairwise PERMANOVA results showed a significant difference between most pairwise comparisons (Adjusted *p* < 0.01, Table S1). Only samples of brood and reproductives were not significantly different. The comparisons with ants (ants vs. soil, ants vs. seeds, ants vs. brood, ants vs. reproductives) show high R^2^ values, indicating that ants explain a substantial proportion of the variance in these comparisons (Table S1). Soil comparisons (soil vs. seeds, soil vs. brood, soil vs. reproductives) show lower R^2^ values, suggesting less variance explained by soil (Table S1).

The similarity percentage (SIMPER) analysis, combined with heatmap visualizations, identified the top 20 bacterial ASVs contributing to the structural composition of bacterial communities within ant and nest soil samples (Fig. 4a, b, Tables S2, S3). Among these, three ASVs (668c7, 94215, and 43e06), were classified within the phylum Firmicutes (class Bacilli) based on the bacterial ASV phylogenetic analysis (Figs. S5, S6). These top three ASVs exhibited markedly higher read abundances in ant samples compared to other sample types (Fig. 4c, d, e), highlighting their distinct association with ants.

**Table 1:** Statistical output of the four linear models that tested the effect of ‘sample type’ on each of four alpha diversity measures (Shannon, Faith’s phylogenetic distance (PD), Pielou’s evenness, and observed ASV’s). Number of samples in each statistical model *N* = 239. ‘Sample type’ was the only explanatory variable in each model and results of the post-hoc test for models in which ‘sample type’ was a significant effect (p-value < 0.05) are shown in Fig. 5

Diversity measure	Sum of squares	DF	F value	*p*-value
Shannon	1363.2	4	691.59	**< 0.0001**
Faith’s PD	6041.3	4	186.99	**< 0.0001**
Pielou’s Evenness	16.924	4	387.15	**< 0.0001**
Observed ASVs	972,137	4	161.61	**< 0.0001**

### All soil samples

**Fig. 6:**
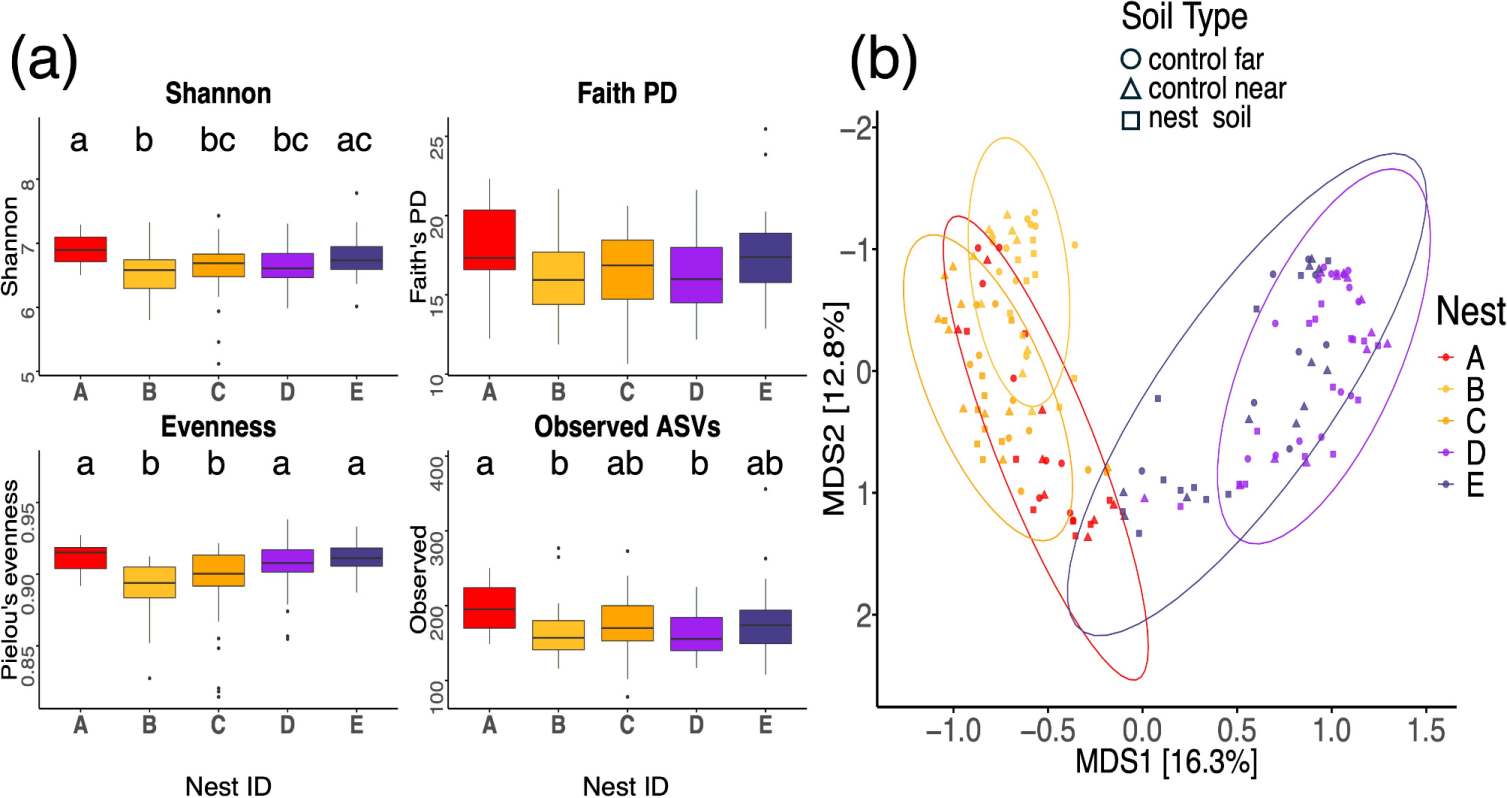
Alpha and beta diversity measures of all soil samples by nest. (**a**) Effect of nest ID (**A**, **B**, **C**, **D**, **E**) on alpha diversity measures (Shannon, Faith’s phylogenetic distance (PD), Pielou’s evenness, and Observed ASVs) of soil samples only. For measures in which nest ID was a significant effect, boxes that do not share a letter are statically significant according to a post hoc Tukey test. (**b**) PCoA plots from a Bray-Curtis dissimilarity distance matrix. Each point is a soil samples with colors corresponding to colony ID and point shape representing soil type (nest soil - squares, control near - triangles, and control far - circles)

**Fig. 7:**
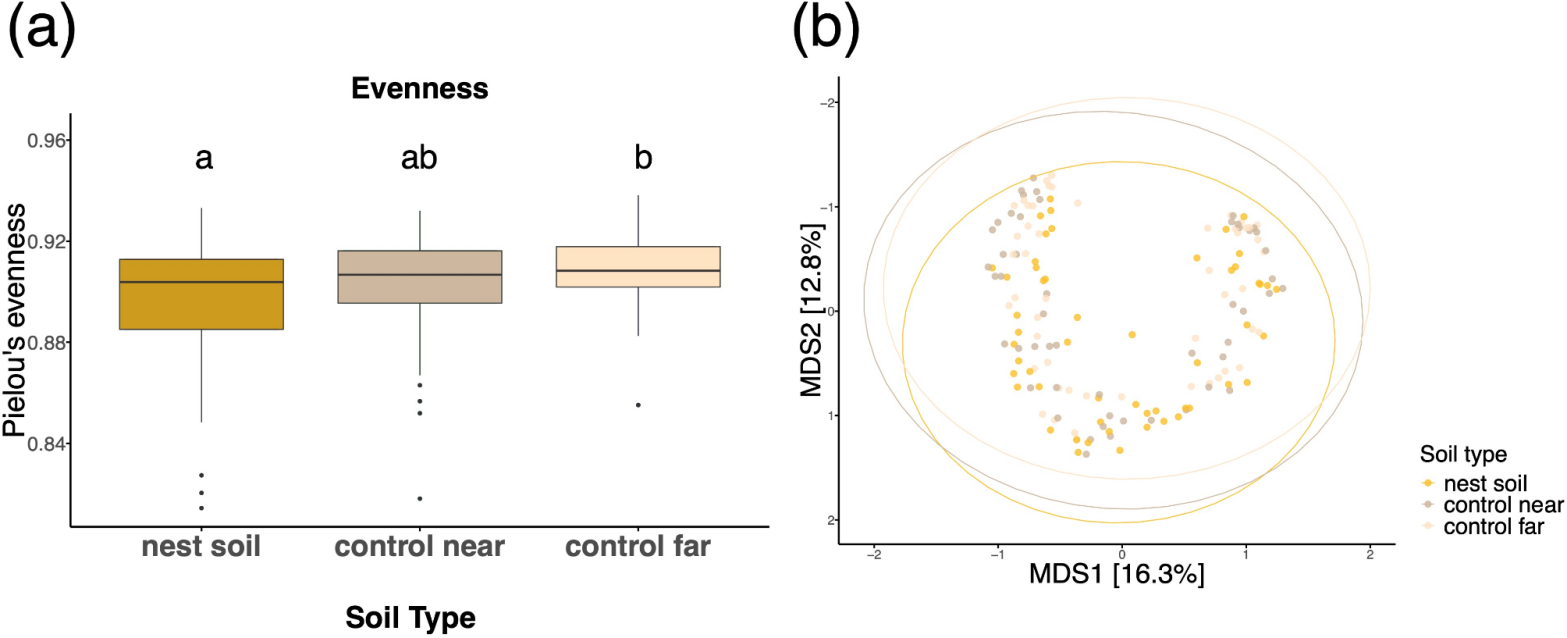
Alpha and beta diversity measures of all soil samples by soil type - same soil samples shown in Fig. 6 with colors corresponding to soil type (nest soil, control near, and control far). Here we show a different view from Fig. 6 to emphasize the effect of soil type. (**a**) Effect of soil type on the alpha diversity measure Pielou’s evenness. Boxes that do not share a letter are statistically significant according to a post hoc Tukey test. (**b**) PCoA plot with Bray-Curtis dissimilarity distance matrix. Each point represents one soil sample and colors correspond to soil type (nest, control near, and control far)

Bacterial alpha diversity of soil from inside and outside the nest differed only for one alpha diversity measure, providing stronger support for the ‘physical environment’ than the ‘social organization’ hypothesis (Table 2). Soil type (nest soil, control near, and control far) significantly impacted only the Pielou’s Evenness index but none of the other alpha diversity measures (Table 2, Fig. 7a). Soil depth did not have a statistically significant effect on any of the alpha diversity measures of the bacterial communities in the soil (Table 2). Finally, nest ID significantly impacted all alpha diversity measures except for Faith’s phylogenetic distance (PD) (Table 2; Fig. 6a).

Bacterial community beta diversity was explained both by soil type (nest soil, control far, control near) and nest ID (A, B, C, D, E). ‘Soil type’ explained 4% of the variation in beta diversity of all soil samples (PERMANOVA: F_DF = 2_ = 12.76, R^2^ = 0.04, p-value = 0.001), this variation was not due to differences in dispersion (PERMDISP: F_DF = 2_ = 2.871, mean sq = 0.006, p-value = 0.061). ‘Nest ID’ explained 25% of the variation in beta diversity of all soil samples (PERMANOVA: F_DF = 4_ = 12.62, R^2^ = 0.25, p-value = 0.001). Thus, both Soil type and nest ID play an important role in determining the variance in the dataset. All pairwise comparisons among soil samples from the five different nests (A, B, C, D, E) were statistically significantly different except for the difference between nests D and E, with adjusted p-values ≤ 0.01 (Table S1). The R^2^ values varied, with the highest being 0.228 and smallest being 0.051 (Table S1). Comparisons of nest soil to control (near and far) were statistically significant but with lower R^2^ values - ranging from 0.012 to 0.042 (Table S1) – and indeed grouping by soil type explains a smaller proportion of the variance (4%) compared to grouping by nest ID (25%). Pairwise PERMANOVA results indicate that control soils - near and far were not significantly different from one another (Table S1).

**Table 2:** Statistical output of the four linear models that tested for the effect of nest, depth, and soil type (chamber soil, control near, and control far) on each of four alpha diversity measures (Shannon, Faith’s phylogenetic distance (PD), Pielou’s evenness, and observed ASV’s). Number of samples in each statistical model *N* = 155. Effects that are statistically significant (p-value < 0.05) are in bold and results of the post-hoc analysis are shown in Figs. 6 and 7

Diversity measure	Effect	Sum of Squares	DF	F value	*p*-value
Shannon	**Nest**	2.675	4	5.524	**< 0.001**
	Depth	0.037	1	0.306	0.581
	Soil type	0.482	2	1.993	0.140
Faith’s PD	Nest	49.53	4	1.885	0.116
	Depth	13.96	1	2.125	0.147
	Soil type	9.65	2	0.735	0.481
Pielou’s Evenness	**Nest**	0.014	4	**9.368**	**< 0.0001**
	Depth	< 0.001	1	0.080	0.777
	**Soil type**	0.005	2	**6.376**	**0.002**
Observed ASVs	**Nest**	19,929	4	**3.340**	**0.012**
	Depth	135	1	0.093	0.764
	Soil type	665	2	0.223	0.801

### Nest soil samples

**Fig. 8:**
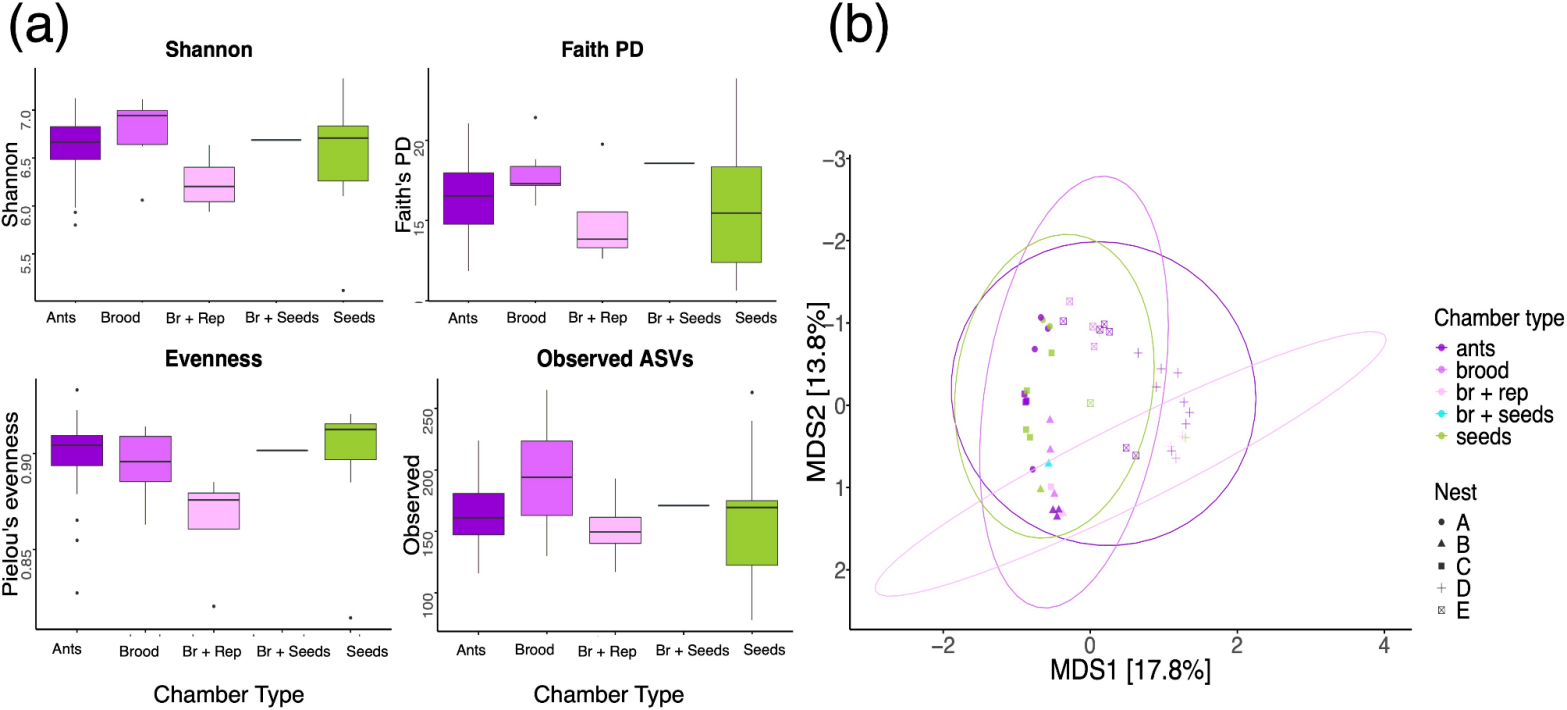
Alpha and beta diversity of soil samples only from inside the nest, by chamber type (ants, brood, brood + reproductive (Br + Rep), brood/seeds (Br + Seeds), and seeds). (**a**) Effect of chamber type on alpha diversity measures (Shannon, Faith’s phylogenetic distance (PD), Pielou’s evenness, and Observed ASVs). Chamber type did not have a statistically significant effect on any of the alpha diversity measures. (**b**) PCoA plot with Bray-Curtis dissimilarity matrix for nest soil by chamber type and nest - colors represent chamber type (ants, brood, brood + seeds, brood + reproductives, and seeds) and point shape corresponds to nest ID

Our comparison across chamber types of bacterial diversity of soil inside the nest did not support either of our two hypotheses. We did not find a significant effect of chamber type or of sample depth on any of the alpha diversity measures (Table 3; Fig. 8a). Similarly, the beta diversity of soil from inside the nest was not explained by chamber type (PERMANOVA: F_DF = 4_ = 1.26, R^2^ = 0.08, p-value = 0.064). However, ‘Nest ID’ had a significant effect on the beta diversity of soil from inside the nest (PERMANOVA: F_DF = 4_ = 5.55, R^2^ = 0.35, p-value = 0.001).

**Table 3:** Statistical output of the four linear models that tested for the effect of nest, depth, and chamber type (based on the content found in the chamber) on each of four alpha diversity measures (Shannon, Faith’s phylogenetic distance (PD), Pielou’s evenness, and observed ASV’s). Number of samples in each statistical model *N* = 53

Diversity measure	Effect	Sum of Squares	DF	F value	*p*-value
Shannon	Nest	2.054	4	3.492	0.165
	Depth	0.252	1	1.710	0.199
	Chamber type	0.834	4	1.417	0.248
Faith's PD	Nest	55.438	4	1.648	0.184
	Depth	5.455	1	0.649	0.426
	Chamber type	34.502	4	1.026	0.407
Pielou’s Evenness	Nest	0.005	4	2.345	0.073
	Depth	0.0008984	1	1.591	0.215
	Chamber type	0.004	4	1.877	0.136
Observed ASVs	Nest	13,147	4	2.393	0.069
	Depth	665	1	0.484	0.491
	Chamber type	6806	4	1.239	0.312

## Discussion

Our study suggests that both social and environmental factors may shape the bacterial communities of V. *andrei* colonies. In support of the physical environment hypothesis (Fig. 1) we found that the bacterial communities of nests in different locations varied significantly across alpha and beta diversity (Fig. 6) and the relative abundance of the nest soil bacterial communities was similar to that of the control soil samples (Fig. 3). In support of the social organization hypothesis, we found that the bacterial communities of the nest contents differed according to biological classification and was different from the bacterial communities of the surrounding soil (Figs. 3 and 5). These differences in bacterial community composition were consistent across the order, class, family, and genus taxonomic levels (Figs. S1, S2, S3, and S4). Furthermore, the beta diversity and evenness of the soil bacterial communities inside the nest was significantly different from the control soil samples (Fig. 8, Table S1). However, the bacterial communities’ diversity of the nest soil did not differ significantly across chambers according to the contents found in them (Fig. 7).

Differences in the bacterial communities’ composition within *V. andrei* nests (biotic and soil samples) provide partial support for the physical environment hypothesis. Overall, the bacterial communities of the biotic content of the nest (ants, reproductives, seeds, and brood) were significantly different from those in the soil inside the nest and the surrounding soil (Figs. 3, 5, S1, S2, S3, and S4). These findings align with previous research indicating that the microbiomes of *Formica exsecta* ants are different from those present in the nest and alpha and beta diversity were lower in ant samples compared to the nest material [84]. However, this work on *F. exsecta* did not differentiate between inside chambers and surrounding soils and only sampled nest material from the top layer of the soil (0–20 cm). Further, our findings are consistent with other work on social insects, such as termites [34], honey bees [31], and ants [37, 38, 85, 86] that show differences in the microbiome of brood, reproductives, and workers. In our study only brood samples were similar to soil samples, according to Faith’s PD diversity measure and Observed ASVs, and reproductives were not significantly different from soil based on Pielou’s evenness. The similarity between brood and soil can be explained by the fact that we did not wash the brood during processing because they would have disintegrated due to the lack of outer protection and contact with harsh chemicals [87]. In addition, we did not wash the seed samples during processing, because we were interested in sequencing the microbes that were found on their exterior, yet we still found significant differences in alpha diversity between the seed and soil samples. Therefore, not washing the brood samples might not be the only explanation for not finding differences between brood and soil samples. The beta diversity of seeds, reproductives, and brood, were all similar (Fig. 5b). This finding might be explained by the fact that brood are the primary consumers of protein [88, 89], which comes from seeds. Protein is required for brood growth, but is not required by worker ants – that do not grow in size after they eclose, and they relay mostly on carbohydrates for energy, and may metabolize lipids from seeds for water [90]. Furthermore, reproductives are most likely recently eclosed, being closer in developmental stage to brood than workers. Thus, it is possible that some bacterial species from seeds are present in the developmental stages that feed on them (brood) and the ants that recently fed on them (reproductives). These findings are consistent with other work on microbiome of honey bees [91] and ants [92], that have highlighted the role of developmental stage on microbiome composition, and with studies that found an impact of diet on microbiome composition of ants and honeybees [93–95].

In further support of the physical environment hypothesis, most alpha diversity measures of the soil bacterial communities inside the nest did not differ from the surrounding soil, either near, or far, from the nest. This result suggests that the bacterial species inside the nest come from the surrounding environment, as seen in nests of arboreal ant species [24, 96]. Indeed, we also found that geographic location impacts the nest bacterial communities. As we predicted, nests in closer proximity had more similar bacterial communities than nests farther apart (Figs. 2a and 6). This similarity can be explained by the similar soil environments because nest bacterial communities’ differences mirrored the physical location of the nests (Fig. 2a), with colonies that were physically closer to each other exhibiting similar alpha and beta diversity (Fig. 6). Such geographic clustering of microbial communities is seen in studies of soil microbiome [97, 98] where microbial communities impact the soil’s physical structure, chemical properties, and water content [97, 99]. Future work might examine how geographical differences in soil microbial composition may affect the behavior of ant colonies and the structure of their nests.

In contrast to the physical environment hypothesis, the beta diversity and evenness of the soil bacterial communities inside the nest was significantly different from the control soil samples (Fig. 8, Table S1). This finding suggests that there are differences in the identity of the taxa observed in soil samples collected from within the nest and soil samples collected approximately one meter away from the nest (Fig. 8, Table S1). These findings align with previous research indicating that ant nests serve as unique microhabitats with distinct microbial activity and soil nutrient composition [51]. However, this previous work used core samples that cut through the nest, and do not distinguish between soil inside the nest and the soil immediately outside the nest chambers - as we did here. Furthermore, they only examined the very top layer of the soil (0–20 cm), whereas, our study did not include samples from the surface of the soil, and most of our samples were from deeper than 20 cm. Interestingly, in contrast with other studies of soil microbiome [27, 100], we did not find a relationship between soil depth and bacterial diversity (Table 3, Figure S7). One possible explanation for this discrepancy could be the unique structure and activity within ant nests, creating microenvironments that sustain higher microbial diversity even at greater depths. Indeed, the digging activity of ants moves soil materials vertically within the nest [41] possibly moving soil-associated microbes along with the moving soil. The unexpected lack of relationship between bacterial diversity and soil depth highlights the complexity of microbial dynamics within ant nests and suggests that additional factors, such as nest architecture and ant activity, may mitigate the typical depth-related decline in microbial diversity.

The social organization hypothesis was supported by the distinct bacterial communities’ composition of each type of biotic nest content (ants, reproductives, seeds, brood). Each of these sample types had different bacterial composition and different alpha diversity (Figs. 3, 5, S1, S2, S3, and S4). The Firmicutes bacterial phylum dominated ant samples whereas Actinobacteria, Proteobacteria, and Firmicutes were more evenly distributed in brood and reproductives (Fig. 3). The presence of Actinobacteria, Proteobacteria, and Firmicutes is typical of herbivorous and omnivorous ant species and larvae [93, 101]. However, Firmicutes is dominant in *V. andrei* adult worker ants, similar to what has been observed in copious predatory ant species such as army ants (Eciton) and bullet ants (*Paraponera clavata*) [101, 102]. Among the bacterial ASVs identified in our study, three Firmicutes (class Bacilli) ASVs specifically stood out due to their markedly higher read abundance across ant samples, irrespective of colony, compared to other sample types (Fig. 4c, d, e). These three ASVs suggest a strong association with ants and may potentially play a role in symbiotic interactions within *V. andrei* ants. Considering past work found that in Azteca ants the microbiome inside chambers matches their content [44], that the chemical signature of nest chambers is determined by their content [45], and that ants use certain nest chambers as latrines [103] it was surprising that we did not find a match between the content of a chamber and the bacterial communities of its soil (i.e., chamber type, Fig. 7). Thus, we did not find support for the idea that spatial division of labor influences and structures the bacterial composition of the nest itself, only that of the biotic content within it. As discussed above, the difference in evenness and beta diversity between nest and control soils suggests that there is some influence of the ants on their nest soil microbiome, however, it does not relate directly to the content of the chambers.

## Conclusions

Our results contribute to a growing body of evidence that social insect nests are intricate ecosystems influenced by both intrinsic and extrinsic factors. Our study highlights the significant roles of both social organization and the physical environment in shaping the microbiome of *V. andrei* colonies. The influence of the surrounding soil bacterial communities on the nest bacterial communities especially underscores the intricate interplay between environmental and social factors in structuring nest microbiome. Thus, future work examining microbial ecology of animals should consider both the physical environment and social organization when studying the animal holobiont.

## Electronic supplementary material

Below is the link to the electronic supplementary material.

Supplementary Material 1: Table S1: Pairwise PERMANOVA results comparing beta-diversity using Bray-Curtis distances between (1) All sample types, (2) All soil types, and (3) Nest Soil.

Supplementary Material 2: Table S2: SIMPER analysis of ant samples. Includes the top 20 ASV taxa which contribute to the observed differences in community structure in the ant samples.

Supplementary Material 3: Table S3: SIMPER analysis of nest soil samples. Includes the top 20 ASV taxa which contribute to the observed differences in community structure in soil samples.

Supplementary Material 4: Figure S1: Relative abundance of bacterial class ordered by sample type: ants, reproductives (R), brood (Br), seeds (S), nest soil, control near soil, control far soil. Each vertical bar is an individual sample with color indicating the bacterial class according to ASV. The sampling depth was 3618 reads.

Supplementary Material 5: Figure S2: Relative abundance of bacterial order organized by sample type: ants, reproductives (R), brood (Br), seeds (S), nest soil, control near soil, control far soil. Each vertical bar is an individual sample with color indicating the bacterial order according to ASV. The sampling depth was 3618 reads.

Supplementary Material 6: Figure S3: Relative abundance of bacterial family ordered by sample type: ants, reproductives (R), brood (Br), seeds (S), nest soil, control near soil, control far soil. Each vertical bar is an individual sample with color indicating the bacterial family according to ASV. The sampling depth was 3618 reads.

Supplementary Material 7: Figure S4: Relative abundance of bacterial genus ordered by sample type: ants, reproductives (R), brood (Br), seeds (S), nest soil, control near soil, control far soil. Each vertical bar is an individual sample with color indicating the bacterial genus according to ASV. The sampling depth was 3618 reads.

Supplementary Material 8: Figure S5: Phylogenetic tree of top 20 bacterial ASVs from ant samples. ASVs are colored by bacterial class.

Supplementary Material 9: Figure S6: Phylogenetic tree of top 20 bacterial ASVs from nest soil samples. ASVs are colored by bacterial class.

Supplementary Material 10: Figure S7: Alpha diversity was not related to chamber depth (normalized by nest depth). Each point represents a chamber in a nest, color indicates soil sample type (nest soil, control near, control far).

## Data Availability

The datasets analyzed during the current study are available in this published article and its supplementary information files and in the repository https://github.com/DAlejandraG/nest-microbes. All sequence data analyzed during this study are available on NCBI BioProject record SUB14655354, https://www.ncbi.nlm.nih.gov/sra/PRJNA1147938.
