# Supplementary figures and images for: Social organization and physical environment shape the microbiome of harvester ants
Source: Anim Microbiome. 2025 Mar 19;7:29. doi: 10.1186/s42523-025-00390-3 (PMC11921602; doi:10.1186/s42523-025-00390-3)

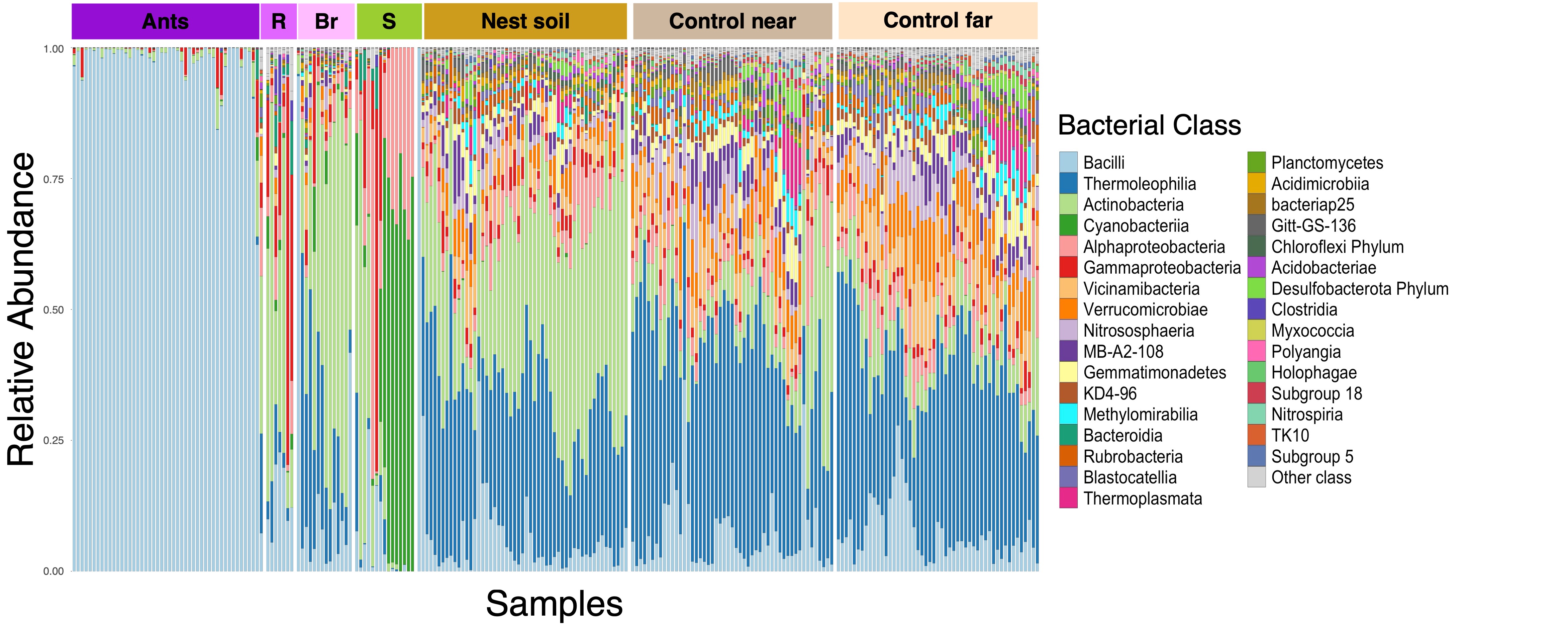

Supplement: Supplementary file 4 — Supplementary Material 4: Figure S1: Relative abundance of bacterial class ordered by sample type: ants, reproductives (R), brood (Br), seeds (S), nest soil, control near soil, control far soil. Each vertical bar is an individual sample with color indicating the bacterial class according to ASV. The sampling depth was 3618 reads. [file 42523_2025_390_MOESM4_ESM.jpg]

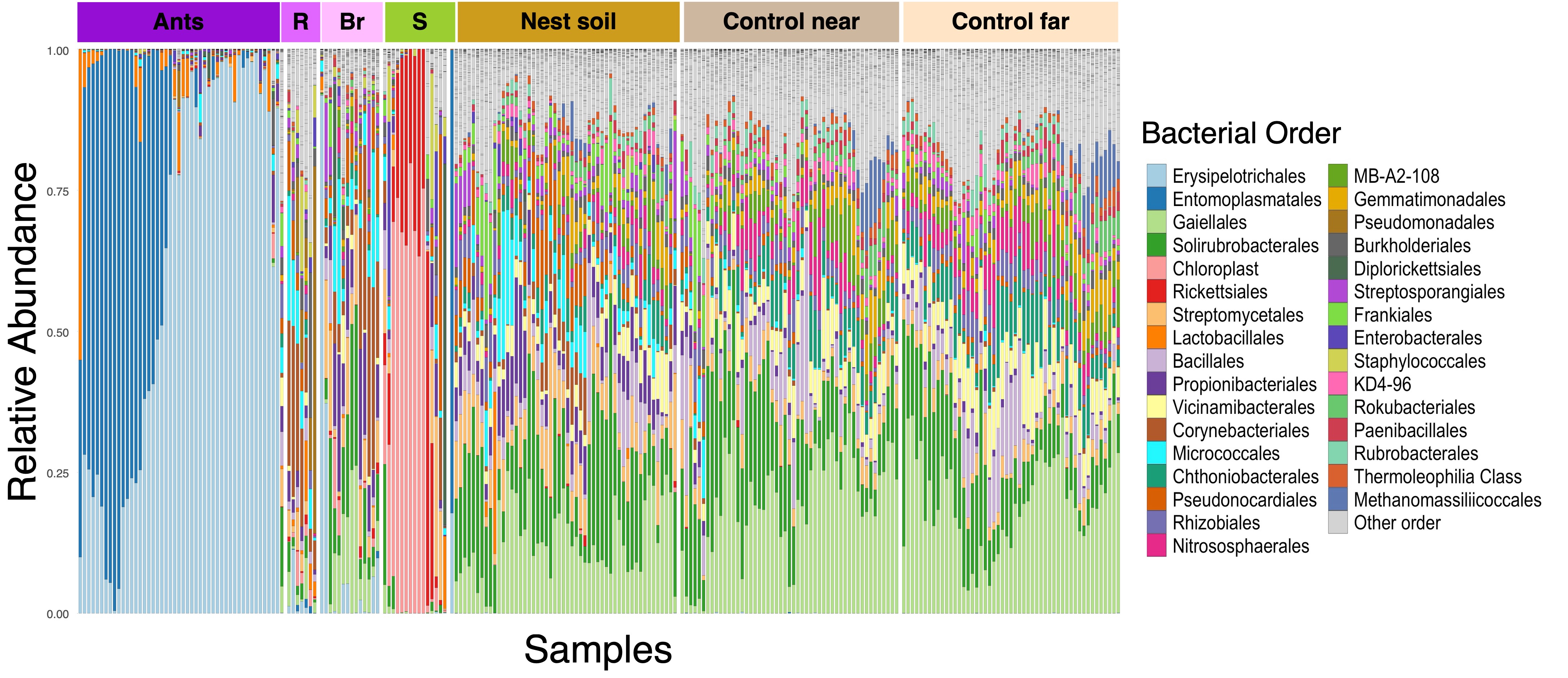

Supplement: Supplementary file 5 — Supplementary Material 5: Figure S2: Relative abundance of bacterial order organized by sample type: ants, reproductives (R), brood (Br), seeds (S), nest soil, control near soil, control far soil. Each vertical bar is an individual sample with color indicating the bacterial order according to ASV. The sampling depth was 3618 reads. [file 42523_2025_390_MOESM5_ESM.jpg]

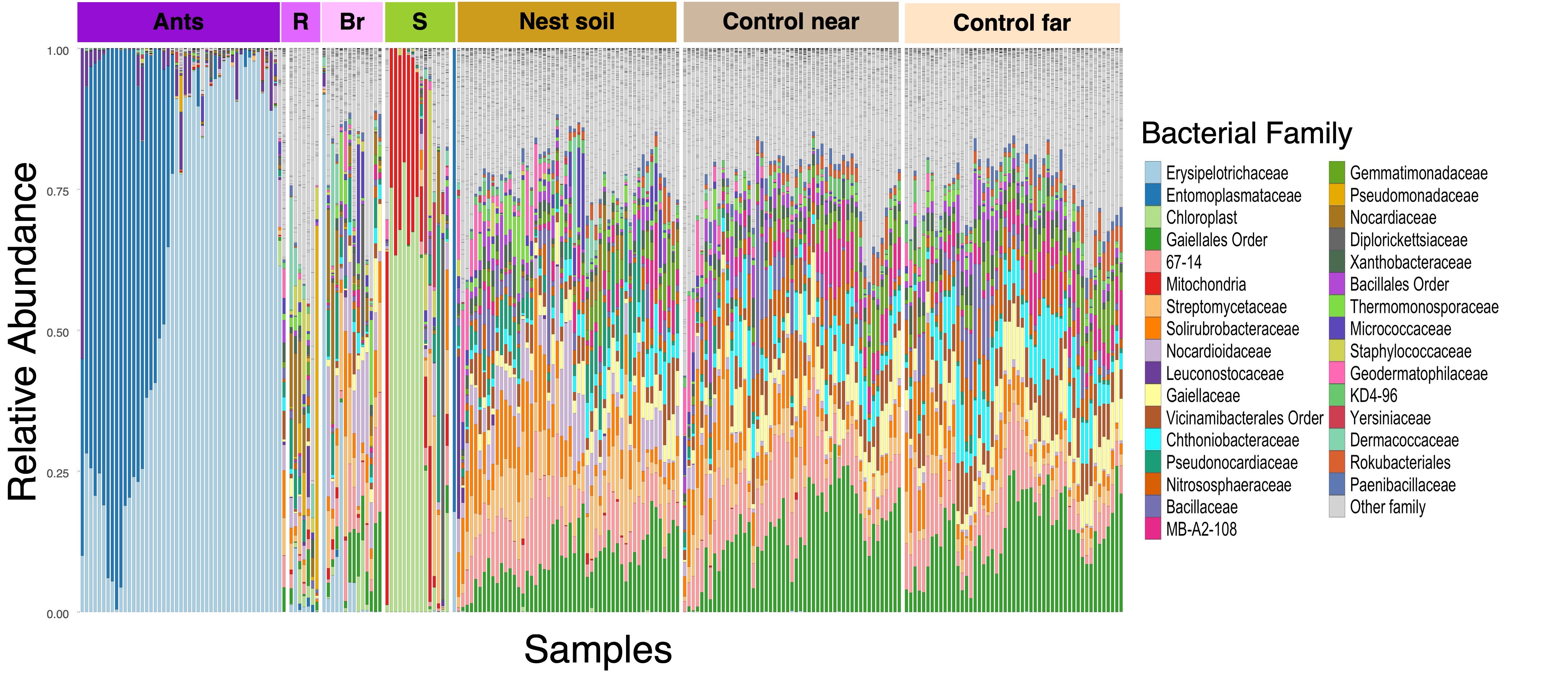

Supplement: Supplementary file 6 — Supplementary Material 6: Figure S3: Relative abundance of bacterial family ordered by sample type: ants, reproductives (R), brood (Br), seeds (S), nest soil, control near soil, control far soil. Each vertical bar is an individual sample with color indicating the bacterial family according to ASV. The sampling depth was 3618 reads. [file 42523_2025_390_MOESM6_ESM.jpg]

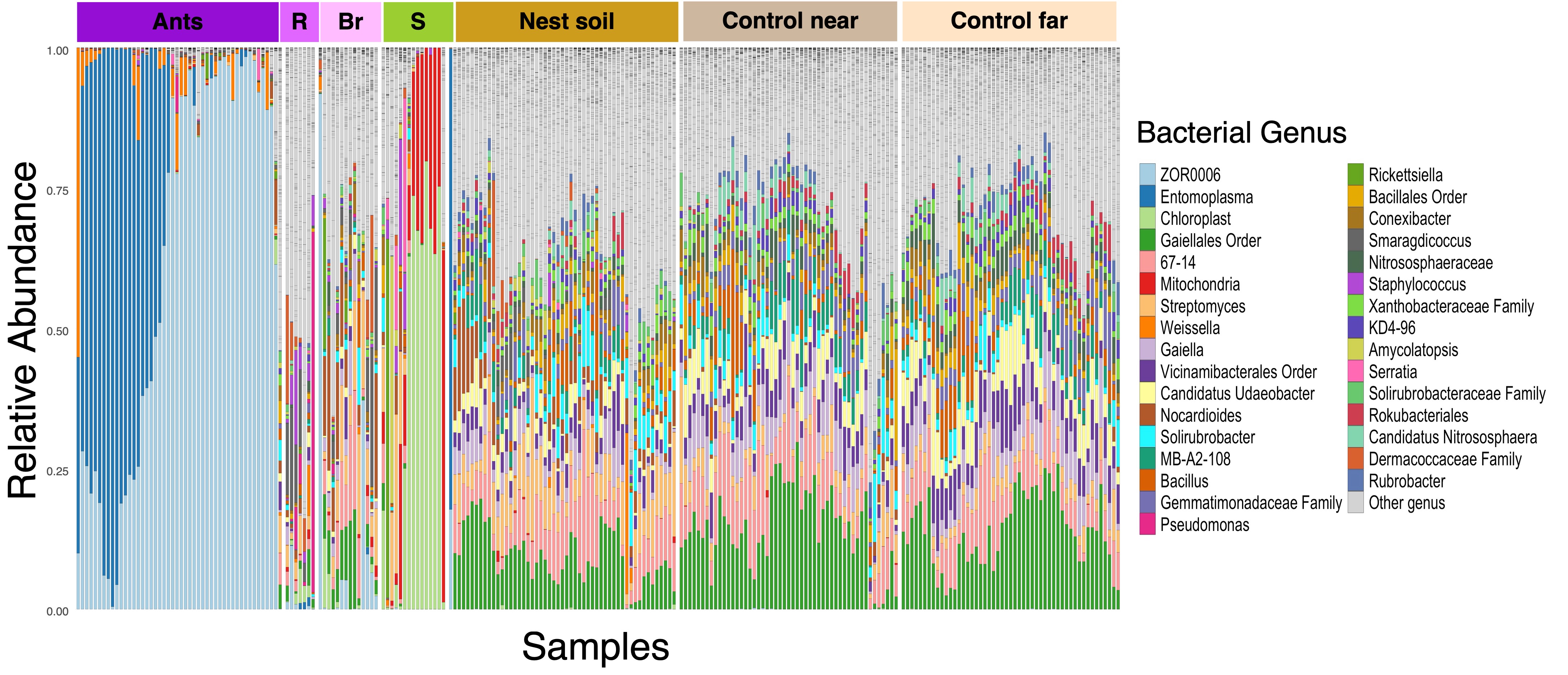

Supplement: Supplementary file 7 — Supplementary Material 7: Figure S4: Relative abundance of bacterial genus ordered by sample type: ants, reproductives (R), brood (Br), seeds (S), nest soil, control near soil, control far soil. Each vertical bar is an individual sample with color indicating the bacterial genus according to ASV. The sampling depth was 3618 reads. [file 42523_2025_390_MOESM7_ESM.jpg]

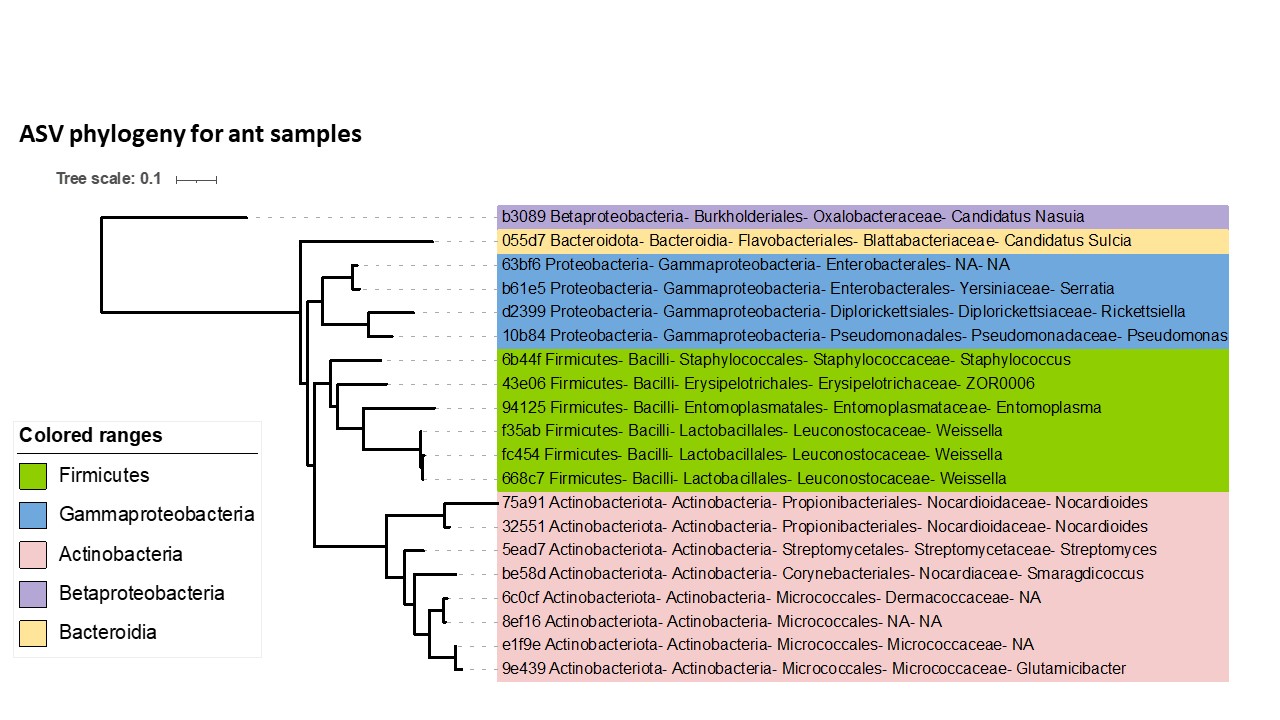

Supplement: Supplementary file 8 — Supplementary Material 8: Figure S5: Phylogenetic tree of top 20 bacterial ASVs from ant samples. ASVs are colored by bacterial class. [file 42523_2025_390_MOESM8_ESM.jpg]

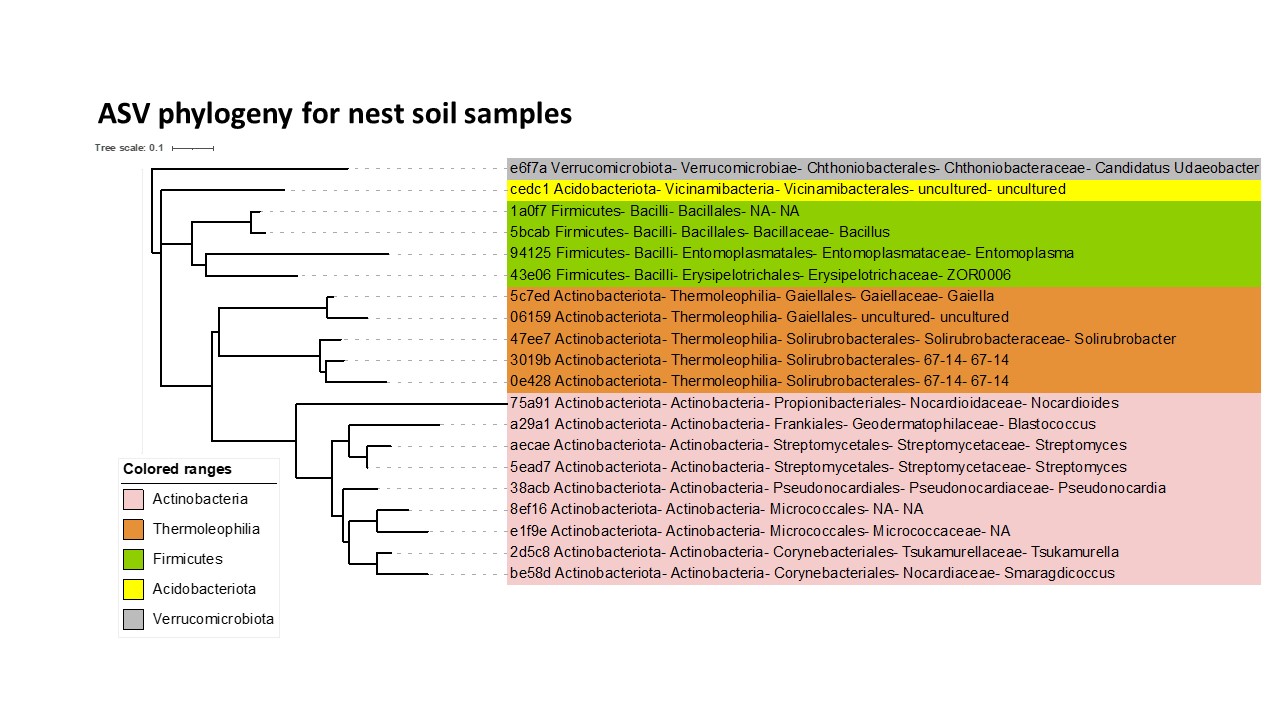

Supplement: Supplementary file 9 — Supplementary Material 9: Figure S6: Phylogenetic tree of top 20 bacterial ASVs from nest soil samples. ASVs are colored by bacterial class. [file 42523_2025_390_MOESM9_ESM.jpg]

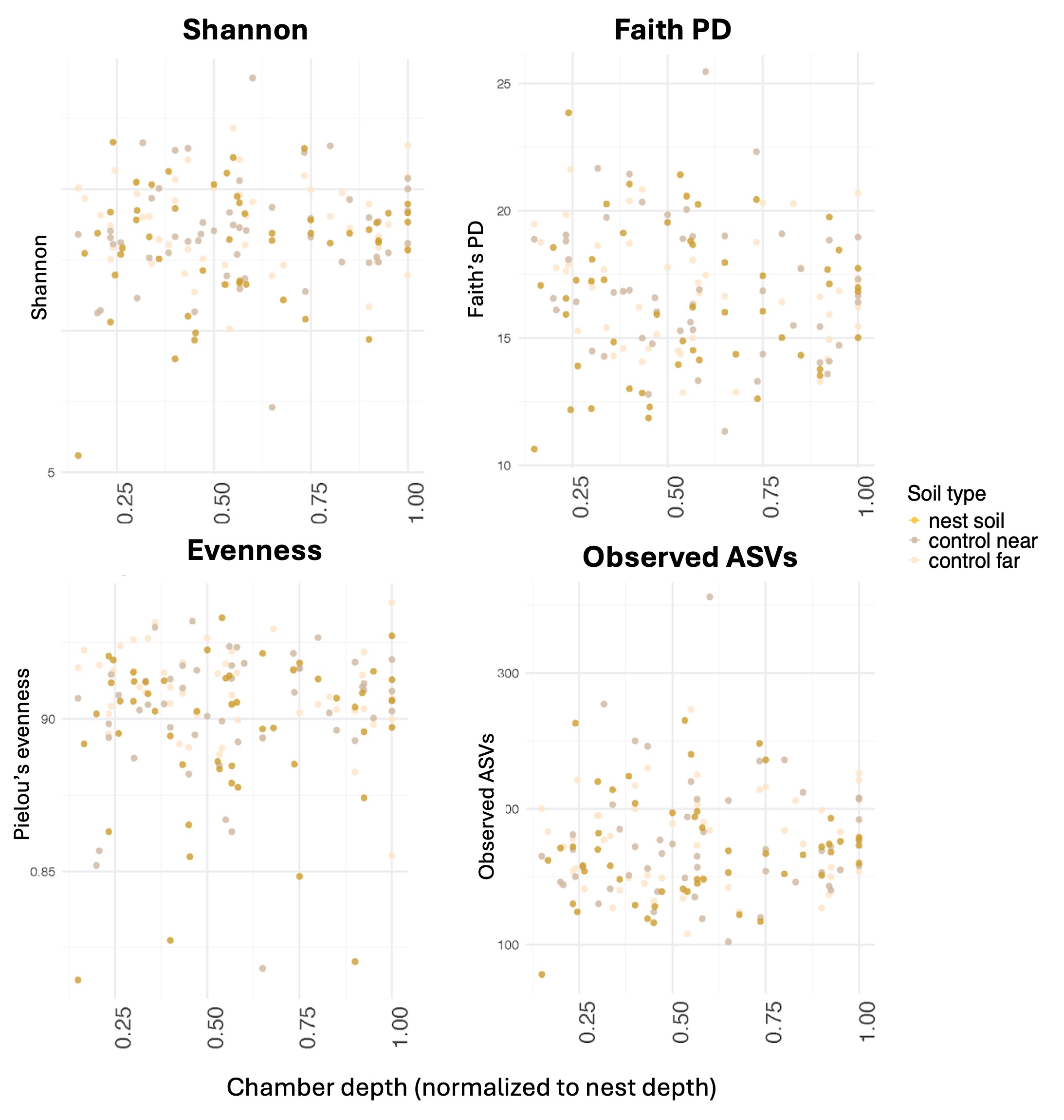

Supplement: Supplementary file 10 — Supplementary Material 10: Figure S7: Alpha diversity was not related to chamber depth (normalized by nest depth). Each point represents a chamber in a nest, color indicates soil sample type (nest soil, control near, control far). [file 42523_2025_390_MOESM10_ESM.jpg]
